# A Deep Learning-Based Framework for Highly Accelerated Prostate MR Dispersion Imaging

**DOI:** 10.3390/cancers16172983

**Published:** 2024-08-27

**Authors:** Kai Zhao, Kaifeng Pang, Alex LingYu Hung, Haoxin Zheng, Ran Yan, Kyunghyun Sung

**Affiliations:** 1 Department of Radiological Sciences, University of California, Los Angeles, CA 92521, USA; 2 Department of Electrical and Computer Engineering, University of California, Los Angeles, CA 92521, USA; kaifengpang@mednet.ucla.edu; 3 Department of Computer Science, University of California, Los Angeles, CA 92521, USA; alexhung96@ucla.edu (A.L.H.); hzheng@mednet.ucla.edu (H.Z.); 4 Department of Bioengineering, University of California, Los Angeles, CA 92521, USA; ranyan@mednet.ucla.edu

**Keywords:** MRI, DCE-MRI, dispersion imaging, prostate cancer, deep learning, transformer

## Abstract

**Simple Summary:**

Nonlinear curve fitting of the pharmacokinetic model to DCE-MRI concentration curves is highly time-consuming. The estimation of highly non-linear dispersion-related parameter in MR dispersion imaging (MRDI) makes the process even more tedious. The fast MRDI (fMRDI) model is proposed to simplify and accelerate the MRDI model by representing the dispersion-applied arterial input function (AIF) as the weighted-sum of a fast and a slow population-based AIFs. A deep learning-based two-stage inference method is proposed to accelerate quantitative MRDI. The deep learning model makes a initial estimation of the parameters directly from the concentration curves and the parameters is then refined by a number of iterative optimization.

**Abstract:**

Dynamic contrast-enhanced magnetic resonance imaging (DCE-MRI) measures microvascular perfusion by capturing the temporal changes of an MRI contrast agent in a target tissue, and it provides valuable information for the diagnosis and prognosis of a wide range of tumors. Quantitative DCE-MRI analysis commonly relies on the nonlinear least square (NLLS) fitting of a pharmacokinetic (PK) model to concentration curves. However, the voxel-wise application of such nonlinear curve fitting is highly time-consuming. The arterial input function (AIF) needs to be utilized in quantitative DCE-MRI analysis. and in practice, a population-based arterial AIF is often used in PK modeling. The contribution of intravascular dispersion to the measured signal enhancement is assumed to be negligible. The MR dispersion imaging (MRDI) model was recently proposed to account for intravascular dispersion, enabling more accurate PK modeling. However, the complexity of the MRDI hinders its practical usability and makes quantitative PK modeling even more time-consuming. In this paper, we propose fast MR dispersion imaging (fMRDI) to effectively represent the intravascular dispersion and highly accelerated PK parameter estimation. We also propose a deep learning-based, two-stage framework to accelerate PK parameter estimation. We used a deep neural network (NN) to estimate PK parameters directly from enhancement curves. The estimation from NN was further refined using several steps of NLLS, which is significantly faster than performing NLLS from random initializations. A data synthesis module is proposed to generate synthetic training data for the NN. Two data-processing modules were introduced to improve the model’s stability against noise and variations. Experiments on our in-house clinical prostate MRI dataset demonstrated that our method significantly reduces the processing time, produces a better distinction between normal and clinically significant prostate cancer (csPCa) lesions, and is more robust against noise than conventional DCE-MRI analysis methods.

## 1. Introduction

**Figure 1:**
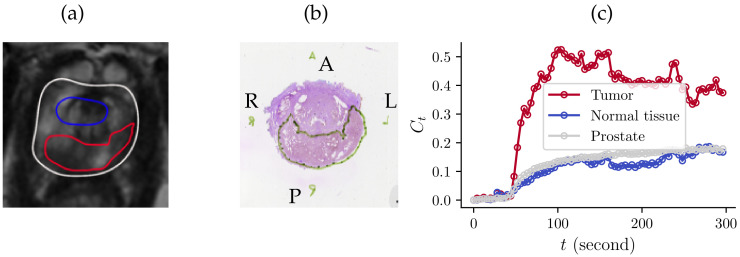
(**a**) T2-weighted MR slice with annotated prostate (gray), normal tissue (blue), and a tumor (red). (**b**) Corresponding histopathology image. (**c**) Concentration curves for the three regions demonstrate different contrast-agent concentrations, $C_{t}\left(t\right)$, in three different ROIs.

Tumor development is associated with the growth of new irregular microvessels, a process known as angiogenesis [1]. The angiogenic microvessels are characterized by leaky vessel walls and, therefore, a high degree of permeability [2]. Increased microvascular density and permeability [3,4] have been reported in several studies to correlate with cancer aggressiveness [5,6]. In the prostate, increased microvascular density and permeability can be characterized by dynamic contrast-enhanced magnetic resonance imaging (DCE-MRI) [7,8,9]. Prostate DCE-MRI acquires a time series of T1-weighted images before, during, and after the injection of a contrast agent (CA, e.g., gadolinium-based contrast agents [10]). After the bolus injection, the CA leaks across the vascular wall to the extracellular extravascular space (EES), resulting in MR enhancement uptakes. The temporal variations in MRI signals provide information about permeability and angiogenesis, which can be quantified as the leakage from the vessel to EES [2,11]. As illustrated in Figure 1, there is a noticeable disparity in tissue-concentration curves between clinically significant prostate cancer (csPCa) and normal prostate tissue. The concentration curve in the tumor exhibits a significantly faster uptake compared to normal tissue, which could be attributed to leaky vascular walls in the tumor [1,12].

Quantitative DCE-MRI analysis aims to extract physiological parameters that reflect the permeability and microvascular density of the underlying tissue. This can be achieved by fitting a pharmacokinetic (PK) model to time-series concentration curves using nonlinear least square (NLLS) curve fitting. The standard Tofts model [10] is a widely used PK model for prostate DCE-MRI. It formulates the tissue concentration, $C_{t}\left(t\right)$, as the convolution of the plasma concentration (or arterial input function, AIF), $C_{p}\left(t\right)$, and the tissue impulse response. The tissue-imposed response is characterized by several PK parameters related to microvascular permeability, such as the forward volume transfer constant, $K^{trans}$, and the flux rate, $k_{ep}$.

The accurate quantification of AIFs is critical to PK modeling [9,13,14,15]. Either the AIF can be measured [15,16] or a population-based AIF can be assumed [14,17,18]. Measuring the AIF necessitates a high temporal resolution [19] and a wide dynamic range [20], which is not always possible in clinical settings [21]. In practice, a population-based AIF is often assumed across patients [14,17,18]. In particular, a line of AIF models [14,22,23] has been proposed to achieve an analytical solution to the convolution in the Tofts model and, therefore, simplify the computation. However, the AIF has a significant impact on the estimated PK parameters [9,16], and location-specific AIFs can improve the estimation [15,16].

Recently, the MR dispersion imaging (MRDI) model [24] has been introduced to characterize the intravascular dispersion of the contrast agent. The quantification of intravascular dispersion inherently yields dispersion-related parameters, which were calculated by applying voxel-wise dispersion to AIFs within the prostate [8,24,25]. Adopting the dispersion-applied AIF in PK modeling is considered to improve the model and parameter estimation precision [8,24,25,26,27,28]. In addition, dispersion-related parameters can be used to identify clinically significant prostate cancer (csPCa) [26,29]. The dispersion-related parameters, either alone [8,24,26] or combined with other PK parameters [25,28], have been shown to be indicative of prostate cancer. In Turco’s study [8], dispersion-related parameters were suggested to outperform other DCE parameters for prostate cancer detection.

The modified MRDI (mMRDI) model [25] simplified the dispersion-applied AIF as a convolution parameterized by a dispersion-related parameter. mMRDI effectively approximates the dispersion-applied AIFs with a reduced number of parameters. Additionally, the dispersion-related parameter in the mMRDI model can be used alongside the volume transfer constant ($K^{trans}$) for prostate cancer diagnosis [25]. However, mMRDI still requires an AIF estimation, and the high computation of MRDI and mMRDI limits their practical usability in prostate DCE-MRI.

Performing voxel-wise curve fitting of the nonlinear PK model can be time-consuming [30,31,32], particularly in prostate DCE-MRI, where a large number of voxels have to be processed in a multi-slice scan. Murase proposed using the simplex method [33] to efficiently fit the PK model, but this method requires a fixed AIF and low sampling interval, limiting its practical application. Moreover, the iterative fitting of a nonlinear PK model is susceptible to noise and initialization [34,35,36,37], and improper initializations could result in suboptimal results. Several workarounds have been used to find proper initializations [25,35]. Dikaios et al. [35] iteratively searched for the initialization values until convergence was achieved. Sung [25] repeated the optimization with ten different initializations and then selected the best results to avoid improper initializations. However, these workarounds introduced extra computation time.

Recently, a line of studies has demonstrated the advantages of using deep learning for fast PK parameter estimation [37,38,39]. Bliesener et al. [37] used deep Bayesian learning to estimate both PK parameters and uncertainty in longitudinal brain DCE-MRI. Ottens et al. [38] compared various deep-learning models for PK parameter estimation in pancreatic cancer detection. Witowski et al. [39] proposed a supervised method that directly localizes lesions from DCE-MRI data for breast cancer detection. These methods either rely on a particular AIF [37,38] or annotated data [38,39], making them less flexible. Directly estimating the parameters of a complicated PK model from noisy DCE-MRI data is challenging, and the estimations of these methods are less accurate [37,38].

**Figure 2:**
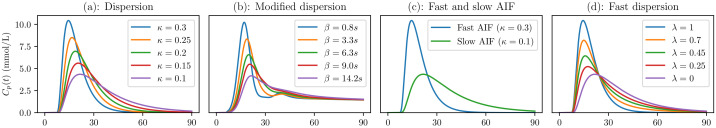
Various dispersion-applied AIFs in MRDI [24] (**a**) and mMRDI [25] (**b**) with different dispersion parameters, κ and λ. Our fMRDI model uses a weighted sum of slow and fast AIFs (**c**) to achieve similar AIFs of different dispersion levels (**d**).

In this paper, we introduce the fast MR dispersion imaging (fMRDI) model, which further simplifies the MRDI model and simulates the dispersion-applied AIF with the weighted sum of AIFs of different dispersion levels. An AIF with greater dispersion exhibits a slow uptake, and an AIF with less dispersion has a fast uptake [25]. Therefore, fMRDI can effectively account for voxel-wise AIFs with various dispersion levels, yielding estimates of the intravascular dispersion-related parameter. As shown in Figure 2, the weighted-sum AIF (Figure 2d) essentially mimics various dispersion levels in MRDI and mMRDI (Figure 2a,b) in a simple form.

The benefits of fMRDI are twofold. First, it resembles intravascular dispersion in a simpler form, making it easier to optimize and less computational. Second, as suggested by [8,24,25], the dispersion parameter λ can be used along with other pharmacokinetic (PK) parameters to differentiate between csPCa and normal tissue. Experiments on clinical prostate DCE-MRI data showed that λ improves the overall csPCa detection accuracy (see Section 3.2).

To accelerate the time-consuming NLLS while maintaining its accuracy, we propose a deep-learning-based, two-stage framework for PK parameter estimation. In particular, we used a transformer-based [40] deep neural network to achieve a coarse estimation of the PK parameters, which serve as the initializations of subsequent NLLS. With the coarse estimation as the initialization, the subsequent NLLS converges in a few steps, significantly reducing the iteration steps compared to plain NLLS. Our network is trained with synthetical DCE-MRI concentration curves. We also designed two data-preprocessing modules, the time series pyramid, and sinusoidal normalization, to improve the robustness of our model against the noise in DCE-MRI dynamic images.

We conducted experiments on our in-house clinical DCE-MRI dataset of 182 patients who underwent prostate MRI prior to radical prostatectomy. We used linear discriminant analysis (LDA) to test the precision of csPCa detection using PK parameters from different methods. Additionally, we experimented with digital reference objects (DROs) to test the quality and robustness of different fitting methods. The experimental results show that our fMRDI model produces PK parameters with high contrast between csPCa and normal tissue. Our method is significantly faster and more robust against noise compared to NLLS. In summary, our method enjoys the following favorable properties:

1. The fMRDI model resembles intravascular dispersion with a simple linear combination of slow and fast AIFs, which is easier to optimize and requires less computation.
2. The dispersion parameter in our fMRDI model can be used to differentiate csPCa from normal tissue and improve the overall performance of csPCa identification (Section 3.2).
3. The two-stage estimation framework is fast, accurate, flexible, and more robust against noise and initializations. It does not restrict the form of the AIF or the sampling interval. It operates significantly faster than NLLS and achieves more accurate fitting results.

The rest of this paper is organized as follows. Section 2.1 briefly reviews the standard Tofts model, as well as the MRDI and mMRDI models, and then it introduces our fast MRDI model. Section 2 systematically presents our deep learning-based framework for PK parameter estimation. Section 2.6 outlines the clinical DCE-MRI data used in our experiments. Section 3 reports and analyzes the experimental results and makes comparisons against other methods. Section 4 and Section 5 examine the motivation and background context of our study, discusses potential limitations and areas for future work, and presents concluding remarks.

## 2. Methods and Materials

### 2.1. From Tofts Model to Fast MRDI Model

We first review the classical Tofts PK model, the modified MRDI model, and our fast MRDI model.

#### 2.1.1. The Tofts Model

In the classical Tofts model [10], the tissue contrast agent (CA) concentration, denoted as $C_{t}$, is represented by the convolution of the plasma CA concentration, also known as arterial input function (AIF), denoted as $C_{p}$, and the tissue impulse response:

(1) $$C_{t}\left(t\right)=\int_{0}^{t}\underset{\mathrm{AIF}}{\underset{︸}{C_{p}\left(\tau -t_{0}\right)}}\cdot \underset{\mathrm{impulse}\;\mathrm{response}}{\underset{︸}{K^{trans}e^{-k_{ep}\cdot \left(t-\tau \right)}}}d\tau ,$$

where $t_{0}$ is the bolus arrival time. The tissue impulse response, $K^{trans}e^{-k_{ep}\cdot t}$, is characterized by a few PK parameters, such as the volume-transfer constant ($K^{trans}$, measured in min^−1^) and the rate constant ($k_{ep}$, measured in min^−1^), that are relevant to tissue perfusion and permeability. $K^{trans}$ and $k_{ep}$, which measure the CA wash-in and wash-out, are commonly associated with csPCa, as indicated by studies such as those of Fütterer et al. [41], Kuenen et al. [24], and Sung et al. [25]. They can enhance lesion visibility, according to the Prostate Imaging Reporting and Data System (PI-RADS) [7].

The classical Tofts model assumes that the intravascular contribution to the measured MR signal is negligible, and constant population-averaged AIFs [17,18,20,38,42] are often used for PK modeling. A recent work [38] assumed an exponential-based AIF obtained from [42] so that the convolution in Equation (1) could be analytically expressed and the estimation could be simplified. However, assuming a constant AIF across all patients is not accurate because AIFs may vary across patients or locations within the same patient.

#### 2.1.2. MRDI and mMRDI: Dispersion-Applied AIFs

The MR dispersion model [24] considered the intravascular dispersion in the prostate and, therefore, characterized location-specific AIFs within the prostate. In particular, the dispersion-applied AIF is formulated as follows:

(2) $$C_{p}{\left(t\right)}^{MRDI}=\alpha \sqrt{\frac{\kappa }{2\pi (t-t_{0})}}\exp\left(\frac{-\kappa {\left(t-t_{0}-\mu \right)}^{2}}{2(t-t_{0})}\right)$$

where κ ($s^{-1}$) is the dispersion parameter, μ is the average transit time from the injection site to the detection site, and α is the integral of $C_{t}$.

In the modified MRDI (mMRDI) model [25], the dispersion-applied AIF is formulated as the convolution of a vascular transport function:

(3) $$C_{p}^{mMRDI}=C_{p}\left(t\right)⊗\frac{1}{\lambda }e^{-\frac{t}{\lambda }}$$

where $C_{p}\left(t\right)$ is a population-based AIF and λ is the dispersion coefficient, (e.g., the larger the λ, the larger the dispersion). mMRDI characterizes intravascular dispersion with a simpler formulation and improves practical usability.

However, the convolution in Equation (3) is nonlinear, and the dispersion parameter λ in the denominator typically makes optimization more challenging. This is because the denominator introduces nonlinearity and discontinuity, which can complicate and destabilize the optimization process [43].

To address optimization instability, mMRDI [25] repeatedly performed NLLS with various initializations and selected the best fit for each voxel. This process can be very tedious, and it takes hours to process a patient.

#### 2.1.3. fMRDI: Fast MRDI Model

To simplify the formulation and ease the optimization, we propose the fast MRDI (fMRDI) model, which represents dispersion-applied AIF as the weighted sum of two AIFs of different dispersion levels.

(4) $${\bar{C}}_{p}^{fMRDI}=\lambda C_{p}^{1}\left(t-t_{0}\right)+\left(1-\lambda \right)C_{p}^{2}\left(t-t_{0}\right),$$

where $C_{p}^{1}$ is the ‘fast’ AIF with less dispersion, and $C_{p}^{2}$ is the ‘slow’ AIF with greater dispersion. λ∈[0,1] is the intravascular dispersion parameter balancing the summation. To achieve AIFs of different dispersion levels, we used the dispersion-applied AIF ($C_{p}^{MRDI}$ in Equation (2)) with $\kappa =0.3\;\;s^{-1},\mu =10$ s as the fast AIF ($C_{p}^{1}$), and $\kappa =0.1\;\;s^{-1},\mu =20$ s as the slow AIF ($C_{p}^{2}$). The two bases were selected according to the distribution of the two parameters in our dataset. Specifically, we calculated μ and κ using the MRDI model on our dataset, and we chose $\kappa =0.3\;\;s^{-1},\mu =10$ s and $\kappa =0.1\;\;s^{-1},\mu =20$ s because more than 99% of the voxel parameters fell within this range. Figure 2a,b,d demonstrate various dispersion-applied AIFs in MRDI [24], mMRDI [25], and our fMRDI model. Figure 2c shows the slow and fast AIFs in fMRDI. Figure 2d shows that the AIFs with various dispersion factors can be similarly achieved using fMRDI with different λ parameters.

As per previous studies [8,25], AIFs in csPCa exhibit lower dispersion levels and faster uptakes, whereas the AIFs of normal tissue have higher dispersion levels and slower uptakes. Therefore, λ is close to 1 in csPCa and close to 0 in normal tissue. This is verified by the results in Figures 11, 13 and 14.

With the simplified dispersion-applied AIF, we can derive the fast MRDI model by substituting Equation (4) for Equation (1):

(5) $$C_{t}\left(t\right)=\int_{0}^{t}\underset{\mathrm{weighted}\text{-}\mathrm{sum}\;\mathrm{AIF}}{\underset{︸}{\left(\lambda C_{p}^{1}+\left(1-\lambda \right)C_{p}^{2}\right)}}\cdot K^{trans}e^{-k_{ep}\left(t-\tau \right)}d\tau ,$$

The derivation of Equation (5) is similar to Equation (4) in [8]. The fMRDI model in Equation (5) provides an assessment of the microvascular architecture using the dispersion parameter λ and of microvascular permeability using $K^{trans}$ and $k_{ep}$.

For simplicity of notation, we will refer to the fast MRDI model as follows:

(6) $$C_{t}=\mathrm{fMRDI}\left(P\right)$$

where $P=\left\{K^{trans},k_{ep},t_{0},\lambda \right\}\in R_{+}^{4}$ are the PK parameters to be estimated. The PK parameter estimation from DCE-MRI data is essentially the reverse of Equation (6), which estimates *P* from $C_{t}$.

**Figure 3:**
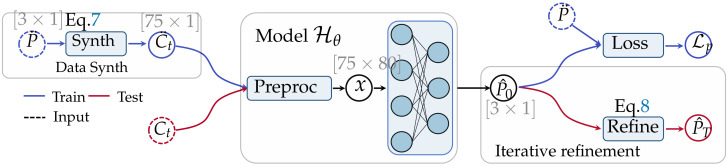
The overall workflow of our proposed method for PK parameter estimation. Circles represent the processed data, and the shape of the data is indicated near each circle. In training, the random PK parameter *P* is sampled to synthesize the training data, $C_{t}$. After preprocessing, the data are fed into the neural network, and the estimated parameter ${\hat{P}}_{0}$ is compared against *P* for loss computation. In testing, the model takes clinical DCE-MRI concentration curves, $C_{t}$, as input, and the subsequent ‘iterative refinement’ takes ${\hat{P}}_{0}$ as the starting point of the iteration and refines the initial estimation with NLLS.

We introduce our deep learning-based framework for PK parameter estimation. In Section 2.2, we introduce our overall pipeline, including training and inference workflows. Then, we describe how we synthesize data for model training in Section 2.3. Section 2.4 provides a brief description of the model architecture and training procedure. And finally, we introduce two novel data-preprocessing modules, sinusoidal normalization and time series pyramid, in Section 2.4.2. The overall workflows are illustrated in Figure 3.

### 2.2. Overall Workflows

Our overall workflows are illustrated in Figure 3. In general, there are three key components in our framework: a data synthesis module, the neural network (NN), and iterative refinement. During training, the data synthesis module synthesizes the training curves ${\ddot{C}}_{t}$ for the NN. The NN takes the synthetical data ${\ddot{C}}_{t}$ as input and predicts the initial coarse estimation ${\hat{P}}_{0}$, which is then compared against the randomly sampled parameter $\ddot{P}$ that was used for data synthesis. There are two crucial preprocessing modules in the neural network model that will be described in detail in Section 2.4.2. During inference, the model takes clinical concentration curves as input and produces the initial estimation of the PK parameters, ${\hat{P}}_{0}$, which is then refined via iterative curve fitting. The final PK parameters after iterative refinement are denoted as ${\hat{P}}_{T}$, where T=20 is the number of iterations.

### 2.3. Training Data Synthesis

Let $C_{t}\in R_{+}^{L}$ be the time-series DCE-MRI data, where *L* is the length of the time series (L=75 in our case). Our model learns a mapping from the time series to the PK parameters:

$$P=H(C_{t})$$

where $H\left(\cdot \right):R_{+}^{L}\to R_{+}^{4}$ is our model, which learns to reverse Equation (6). To train such a model, we need the pairwise training datasets $\left\{\left(C_{t}^{1},P^{1}\right),\left(C_{t}^{2},P^{2}\right),...,\left(C_{t}^{N},P^{N}\right)\right\}$. Instead of collecting training datasets from clinical scans, we designed a data synthesis framework to generate unlimited training data for our neural network. A similar synthesis process was also used in [38,44] for model training. In general, the data synthesis module is composed of three steps.

1. Sample random PK parameters $K^{trans},k_{ep},t_{0}$, and λ from designated distributions.
2. Synthesize smooth time series using the fMRDI formulated in Equation (5).
3. Add Gaussian noise to the smooth time series to close the gap between synthetical and real data.

**Figure 4:**
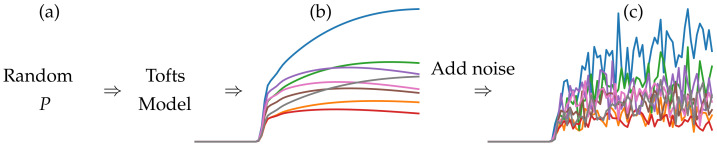
The pipeline of concentration-curve synthesis. (**a**) random PK parameters are sampled to synthesize smooth curves (**b**). (**c**) noise is added to the smooth curves to simulate the real cases.

**Figure 5:**
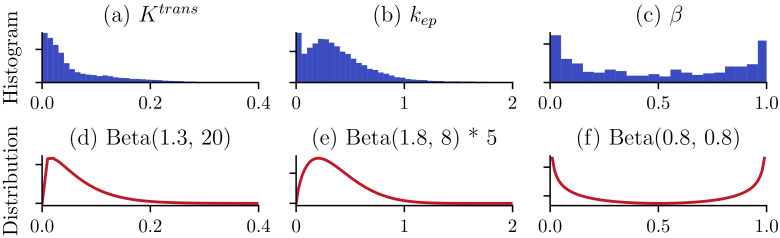
Histograms of pharmacokinetic parameters (**top**) and distributions used for data synthesis (**bottom**).

**Figure 6:**
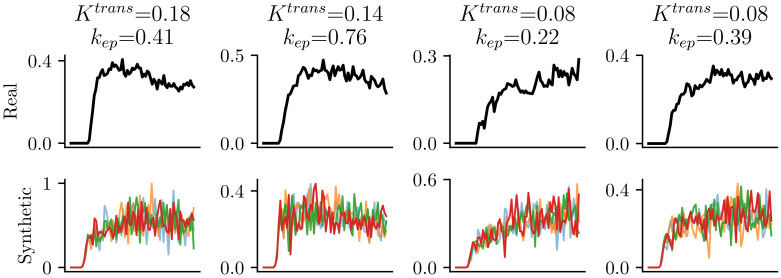
Real (**top**) and synthetic concentration curves (**bottom**). Curves of each column share the same PK parameters.

The overall pipeline of data synthesis is illustrated in Figure 4, the distributions used to sample PK parameters are shown in Figure 5, and some examples of synthetical and real data can be found in Figure 6.

(1) Sample PK parameters As a statistical model, the predictions from neural networks are highly related to the statistics of the training data. To mimic the statistics of real DCE-MRI data, we first analyzed the PK parameters of clinical DCE-MRI cases. Specifically, we estimated the PK parameters of 20 patients using conventional nonlinear least square (NLLS) curve fitting and then constructed their histograms. As shown in Figure 5 (top), the histograms closely resemble specific beta distributions. Then, we used beta distributions, to sample PK parameters for data synthesis. The beta distributions used for data synthesis are shown in Figure 5 (bottom). $t_{0}$ is sampled from a uniform distribution, U(0,0.1).

(2) Synthesize smooth time series Let $\dot{P}$ be the randomly sampled PK parameter from designated distributions. We synthesized a smooth time series using the fMRDI model defined in Equation (6). Example smooth curves are demonstrated in Figure 4.

(3) Add Gaussian noise In order to close the gap between real and synthetical time series, we added random Gaussian noise to the smooth time series. The data synthesis can be formulated as follows:

(7) $${\ddot{C}}_{t}\left(t\right)=\left\lfloor \mathrm{fMRDI}\left(\dot{P}\right)\times \left(1+N(0,\gamma )\right)\right\rfloor ,$$

where $N\left(0,\gamma \right)\in R^{L}$ is the Gaussian noise with zero mean, and the standard deviation of the noise is randomly sampled to achieve synthetical data with a signal-to-noise ratio (SNR) between 4 and 32. ⌊·⌋ is the rectification operator that rectifies all values less than zero as zero because the DCE-MRI data are always greater than zero. Example noisy synthetical curves are demonstrated in Figure 6.

### 2.4. Model Training Workflow

#### 2.4.1. Model Architecture

**Figure 7:**
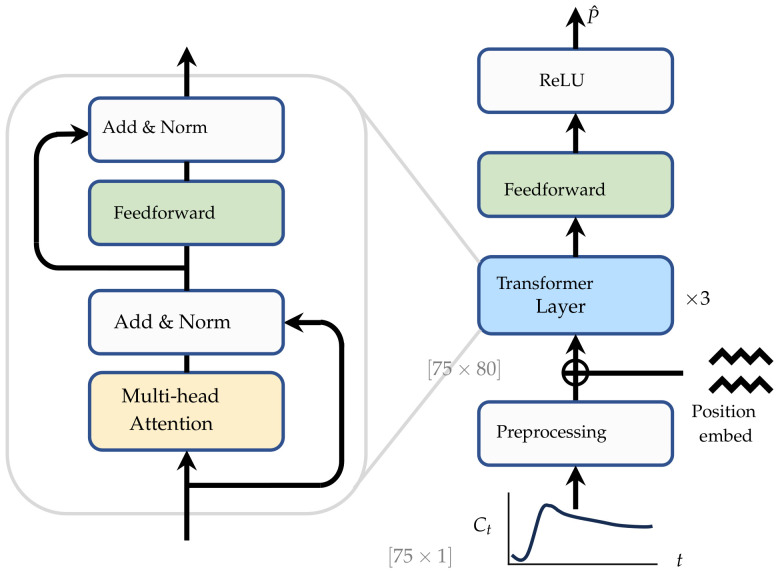
The architecture of the transformer neural network used in our experiments. ReLU activation was used in the last of the network to ensure positive estimations.

We utilized the transformer architecture, which is renowned for its outstanding performance using sequential data. We used a three-layer transformer network, and a similar network has been widely used in computer vision [45] and natural language processing [40]. As shown in Figure 7, the input time-series $C_{t}$ first passes through preprocessing modules, which is detailed in Section 2.4.2, after which the position embedding is added. Afterward, three transformer layers process the high-dimensional inputs, and then a feedforward network produces the predictions. Rectified linear unit (ReLU) activation is used in the end to ensure positive predictions. ReLU is an activation function that retains positive input values while setting negative inputs to zero. The detailed architecture of a transformer layer is illustrated on the left side of Figure 7.

#### 2.4.2. Preprocessing for Robust Neural Networks

The time-series dynamic images are noisy, unnormalized, and presented as one-dimensional time series, posing challenges in training deep neural networks. We propose two crucial data preprocessing modules to stabilize the training process and enhance the model’s performance. The purposes of the two preprocessing modules are threefold:

1. To enhance the model’s robustness against the noise and capture information at various scales.
2. To increase the data dimension and project the one-dimensional time series into high-dimensional space.
3. To normalize the time series data into a fixed range with zero mean and constant variance.

The experimental results in Table 1 demonstrate that the proposed preprocessing modules improve the estimation accuracy.

Without a loss of generality, let $R^{L\times D}$ be the shape of input to each preprocessing module, where *L* is the length of the time series and *D* is the feature dimension (which is one for $C_{t}$). The preprocessing modules process each row of the input independently and generate *N* rows based on a single input row, leading to output data with the shape of $R^{L\times (D\cdot N)}$. *N* is the scaling factor, which means that the module increases the feature dimension of the input by a certain factor.

The scaling factor is set to N=5 in the time series pyramid, and N=16 is used in the sinusoidal normalization. In general, the DCE-MRI time series has a shape of L×1 and will be sequentially processed through the time-series pyramid and the sinusoidal normalization. The data shape after the time-series pyramid is L×5, and after sinusoidal normalization, the shape becomes L×80.

Time series pyramid Our first preprocessing module is the *time series pyramid*, which convolves $C_{t}$ with kernels of different scales to depress the noise and capture information at various contextual scales. This operation draws deep inspiration from the seminal concept of an image pyramid [46] in image processing and computer vision. The image pyramid provides a flexible and convenient multiresolution format that closely resembles the multi-scale processing in the human perceptual system.

We construct the time series pyramid, similar to the image pyramid, by convolving the original time series with Gaussian kernels of different variances (sigmas). Let $K^{\sigma }$ be the Gaussian kernel, where σ is the variance of the kernel, and 2σ+1 is the kernel size. The convolved time series is denoted as $C_{t}^{\sigma }=C_{t}⊗K^{\sigma }.$ We construct the time series pyramid by stacking several convolutions. Let *N* be the number of Gaussian kernels used to construct the pyramid. For arbitrary input $C_{t}\in R{+}^{L\times D}$, where *L* is the length of the time series, and *D* is the feature dimension, the dimension of the pyramid would be $C_{t}\in R{+}^{L\times (D\times N)}$.

**Figure 8:**
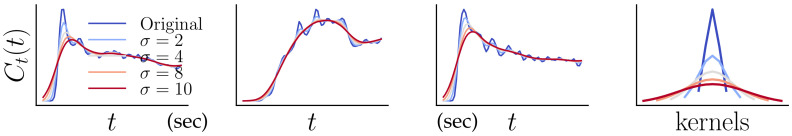
The time series pyramid with different σ and respective Gaussian kernels.

In practice, we used N=5 different kernels with σ=0,2,4,8,10, where σ=0 corresponds to the original signal. The time series pyramid takes raw DCE-MRI data as input (with shape L×1), and the output shape is L×5. Example pyramids and corresponding Gaussian kernels can be found in Figure 8.

Sinusoidal normalization Data normalization is critical to deep neural networks. Normalization techniques rescale input data in a fixed range with zero mean and constant variance, which accelerates the convergence and improves the performance [47,48] of deep neural networks.

In DCE-MRI, the magnitude range exhibits significant variations among time series from different locations. Additionally, the intensity values are highly biased and follow a long-tail distribution. We employ simple sinusoidal functions to normalize the input data into a fixed range of [−1,1]. To normalize the input data while maintaining an injective mapping between the input and output, we utilize a series of sinusoidal functions with different frequencies: $[\sin\left(1\omega_{0}x\right),\cos\left(1\omega_{0}x\right),...,\sin\left(8\omega_{0}x\right),\cos\left(8\omega_{0}x\right)].$ where $w_{0}=2\pi /100$ is the fundamental frequency. The sinusoidal functions project the input into higher-dimensional spaces and ensure a one-to-one mapping between the input and output. The signals after the sinusoidal functions fall within a fixed range and possess a zero mean with constant variance. The input to sinusoidal normalization is the output of the time series pyramid, which has a shape of L×5. The output of sinusoidal normalization has a shape of L×80.

#### 2.4.3. Model Training

Let $H_{\theta }\left(\cdot \right)$ be our model parameterized by θ. Our model is trained with synthetical data introduced in Section 2.3. Suppose $\left({\ddot{C}}_{t},\ddot{P}\right)$ is the training data pair; the model takes ${\ddot{C}}_{t}$ as input and predicts ${\hat{P}}_{0}$, as shown in Figure 3. Our model is trained by minimizing the $L_{1}$ discrepancy between the estimated PK parameter ${\hat{P}}_{0}$ and the one used for data synthesis $\ddot{P}$: $L=\left|\hat{P}-\ddot{P}\right|$, where L is the training loss. The loss is computed separately for each sample (concentration curve) and then averaged across the batch. We use the ADAM algorithm to optimize the neural network. The model is trained for 100,000 iterations with a fixed learning rate of $10^{-4}$ and a batch size of 256.

### 2.5. Model Inference Workflow

#### 2.5.1. From MRI Signal to CA Concentration

Before any pharmacokinetic analysis can take place, the CA concentration, $C_{t}$, has to be calculated from the MRI signal enhancement. $T_{1}$ is reduced from the pre-contrast value $T_{10}$ by the presence of CA: $\frac{1}{T_{1}}=\frac{1}{T_{10}}+r_{1}C$, where $r_{1}$ is the relaxivity, and usually an in vitro value of 4.5 $L\times {\mathrm{mmol}}^{-1}\times s^{-1}$ is used. The CA concentration $C_{t}$ can be expressed as $C_{t}\left(t\right)=\left(\frac{1}{T_{1}\left(t\right)}-\frac{1}{T_{10}}\right)/r_{1}$, where $T_{10}$ is the precontrast $T_{1}$ that can be obtained from the variable flip-angle method [25,49].

#### 2.5.2. Initial Coarse Estimation

Given the CA concentration curve, $C_{t}\left(t\right)$, the initial estimation of the PK parameters is made via the neural network: ${\hat{P}}_{0}=H\left(C_{t}\right)$.

#### 2.5.3. Coarse-to-Fine via Iterative Fitting

We refine the initial estimation, ${\hat{P}}_{0}$, with iterative curve fitting. Specifically, we use the square of the residual as the objective and iteratively update ${\hat{P}}_{\tau }$ to minimize the discrepancy between fitting and the observation using gradient descent. The PK parameter ${\hat{P}}_{\tau }$ is updated based on the following rule:

(8) $${\hat{P}}_{\tau +1}={\hat{P}}_{\tau }-\gamma \frac{\partial {\left|\mathrm{fMRDI}\left({\hat{P}}_{\tau }\right)-C_{t}\right|}^{2}}{\partial {\hat{P}}_{\tau }}$$

where τ is the step index, and γ=0.01 is the learning rate (or step size). In our experiments, Equation (8) converges significantly faster than starting from scratch. The running time, starting from the initial estimation and starting from scratch, can be found in Table 2.

### 2.6. Study Population and DCE-MRI Data

Our retrospective study was conducted in compliance with the 1996 Health Insurance Portability and Accountability Act (HIPAA) and approved by the Institutional Review Board (IRB) of the University of California, Los Angeles, with a waiver of the requirement for informed consent. All methods were performed in accordance with the relevant guidelines and regulations.

The study cohort was derived from patients who underwent 3T mpMRI exams prior to robotic-assisted radical prostatectomy at a single academic center between December 2010 and July 2019. Patients with prior radiotherapy or partial prostate resection, and those with technical limitations, were excluded from the study. The complete dataset comprised 182 patients who had whole-mount histopathology (WMHP) conformed with prostate cancer lesions (PCa). Patient-specific, 3D-printed prostate molds were used to hold the surgically excised prostate glands in the same orientation observed in vivo MRI.

All imaging, including T2W, DWI, and DCE-MRI, was performed on several 3T MRI scanners using a pelvic phased-array coil. The DCE-MRI protocol consisted of precontrast T1(T10) mapping and dynamic imaging. The variable flip angle (VFA) imaging was used for T10 mapping for the conversion of signal intensity to contrast agent concentration. With a temporal resolution of 4∼5 s, dynamic 3D images were acquired before, during, and after a single-dose injection of gadopentetate dimeglumine (Magnevist; Bayer, Wayne, NJ, USA) at a dose of 0.1 mmol/kg through an eripheral vein at a rate of 2 mL/s via a mechanical injector. Additionally, 6∼10 precontrast frames (total acquisition time to be around 24∼50 s), and a total of 75 frames were acquired sequentially without a delay between acquisitions. The last frame of precontrast acquisitions was located by searching for the largest gradient concentration curves in the first 15 frames. All concentration values in precontrast acquisitions were set to zero for ease of optimization. A total of 1500 images were acquired, and the dimension of the DCE-MRI data was 160×160×20×75, where 160×160 is the in-plane resolution, 20 is the number of slices, and 75 is the number of frames.

Each prostate MRI scan was reviewed by genitourinary (GU) radiologists (10+ years of clinical prostate MRI reading experience) as part of the standard clinical care, following the PI- RADS v2.1 guideline. All clinically significant PCa (csPCa) lesions were initially annotated on T2-weighted MRI, and later, research fellows (K.Z. and K.P.) supervised by an MRI scientist (K.S. with 15+ years of experience analyzing DCE-MRI) refined the regions of interest (ROIs) on DCE-MRI using MRI and WMHP as references.

## 3. Experiments and Results

We compared our method with conventional NLLS and a deep learning-based method [38]. We used the CNN architecture for the baseline deep learning-based method [38] due to its simplicity and higher performance. For NLLS, we used the trust region-refective algorithm with a step tolerance of $10^{-2}$, a function tolerance of $10^{-3}$, and a minimum gradient change of 0.1. All implementations were based on the PyTorch framework.

### 3.1. Running Time and Quality of Fitting

#### 3.1.1. Running Time and Fitting Errors

We first compared the quantitative fitting errors and running time. Let $\hat{P}$ be the estimated PK parameter; then, we used Equation (6) to get the fitting curves, ${\hat{C}}_{t}=\mathrm{MRDI}\left(\hat{P}\right)$. The fitting quality was quantified according to the squared error between the reconstructed and the original concentration curves: $error={\left|C_{t}-{\hat{C}}_{t}\right|}^{2}$. We measured the per-patient processing time on an Intel(R) Xeon(R) W-2123 CPU@3.60GHz CPU. For each iterative method, we stopped the iteration if the loss reduction was less than 0.5% for five consecutive iterations. The fitting error, average number of iterations, and running time are summarized in Table 2.

In Table 2, ‘NLLS’, ‘NN’, and ‘NN + NLLS refine’ denote different curve-fitting methods, where ‘NN’ denotes the direct estimation of PK parameters with neural networks, and ‘NN + NLLS refine’ denotes our proposed two-stage method that refines the NN estimation with subsequent NLLS iterations. When starting from scratch, NLLS was performed for 240 iterations. When using ‘NN + NLLS’, we ran 20 iterations.

In general, with the same fitting method, such as NLLS, our fMRDI model significantly reduced the fitting error compared to the Tofts model with the Parker AIF. Furthermore, using the same PK model, such as Tofts + Parker, the two-stage ‘NN + NLLS’ fitting method significantly reduced the running time by a factor of more than four while achieving a lower fitting error. These results demonstrate the strong efficiency and accuracy of our method in PK modeling.

**Figure 9:**
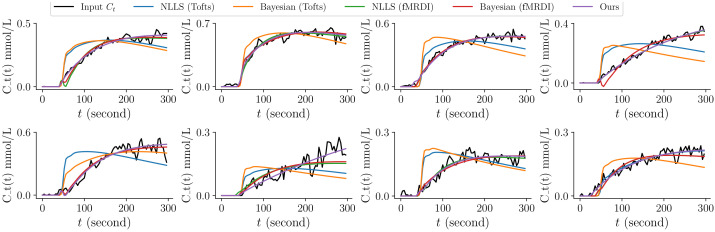
Example fitting results of different methods. Methods with Parker AIF do not fit data with slow uptakes well. fMRDI model achieves the best overall fittings that match the data points the best.

The running time and fitting error are summarized in Table 2, and some example fitting results are in Figure 9.

We also tested the fitting error with different components: the *fMRDI model*, the *NN inference + iterative refinement pipeline*, and the two *preprocessing modules*. The results in Table 1 clearly demonstrate that each individual component contributes incrementally to the quality of fitting and plays a role in our method. The results in Table 1 were confirmed with the paired *t*-test with p<0.01 to verify the effectiveness of each component.

#### 3.1.2. Compared with MRDI and mMRDI

We compared the fitting quality of our method with MRDI and mMRDI and evaluated their robustness. In particular, we fit our fMRDI, MRDI [24], and mMRDI [25] models with NLLS and various numbers of random initializations. For each model, we repeated NLLS with different random initializations and then selected the best fitting for each voxel. The model is considered more stable if it achieves higher performance with fewer repeats.

**Figure 10:**
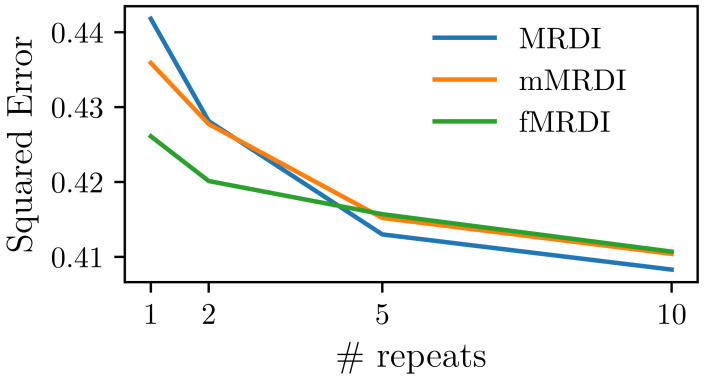
The squared error of fitting with different numbers of repeats. We performed NLLS fitting various times with random initializations and then picked the best fitting for each voxel.

As shown in Figure 10, fMRDI is more stable and less susceptible to initialization, as it achieves a lower error with fewer repeats. This is because the linear weighted sum AIF is much simpler than MRDI and mMRDI, making it easier to optimize. When a saturated number of repeats is performed, the three models perform similarly, with MRDI slightly better than the other two, as MRDI has more free parameters.

### 3.2. csPCa Lesions with $K^{trans}$

We compared PK parametric maps derived from different methods and evaluated the contrast of PK parameters in csPCa and normal tissue. Both the conventional NLLS and the baseline deep learning-based method with exponential AIF [38] were included for comparison. We first visualized different parametric maps and then quantitatively compared the parameter values in csPCa and normal tissue. Linear discrimination analysis (LDA) was lastly performed to quantitatively evaluate the discrimination of csPCa from normal tissue.

Since the volume-transfer constant ($K^{trans}$, min^−1^) [7] and the dispersion parameter λ [8,24,25] were considered to be indicative of csPCa, we only visualized $K^{trans}$ and λ.

#### 3.2.1. Qualitative Visualization of $K^{trans}$ Maps

**Figure 11:**
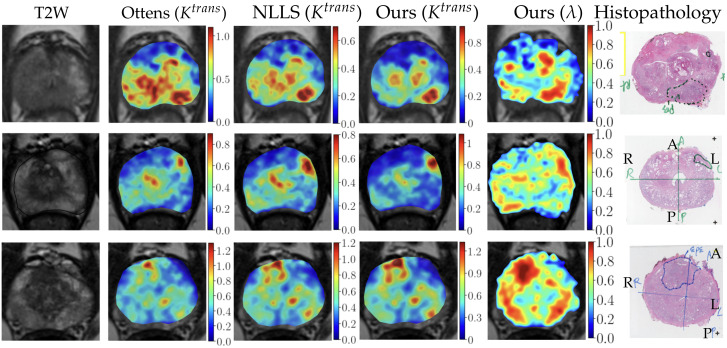
Visualization of PK parametric maps generated using different methods and the beta $K^{trans}$ maps proposed in our study. The T2-weighted images are used as a background, and corresponding histopathological images are provided in the right-most column for reference to identify the location of the lesion.

Figure 11 displays several exemplary $K^{trans}$ maps (1–5 columns), along with corresponding histopathological maps (last column) that indicate the tumor locations. ‘Ours’ represents the fMRDI model with the *NN+refine* fitting method. Each row represents maps of the same patient generated using different methods. Since the tumor spans across multiple slices, we visualized the maximum intensity projection (MIP) of two to five consecutive slices. The specific number of slices in MIP varies among patients. As shown in Figure 11, our $K^{trans}$ maps generally have better contrast in identifying csPCa lesions and are less noisy than other methods.

#### 3.2.2. Quantitative Comparison of Tissue Contrast

In addition to qualitative visualization, we quantitatively analyzed the contrast between the csPCa and normal tissue. The quantitative analysis was conducted on the ROI-averaged parametric maps.

**Figure 12:**
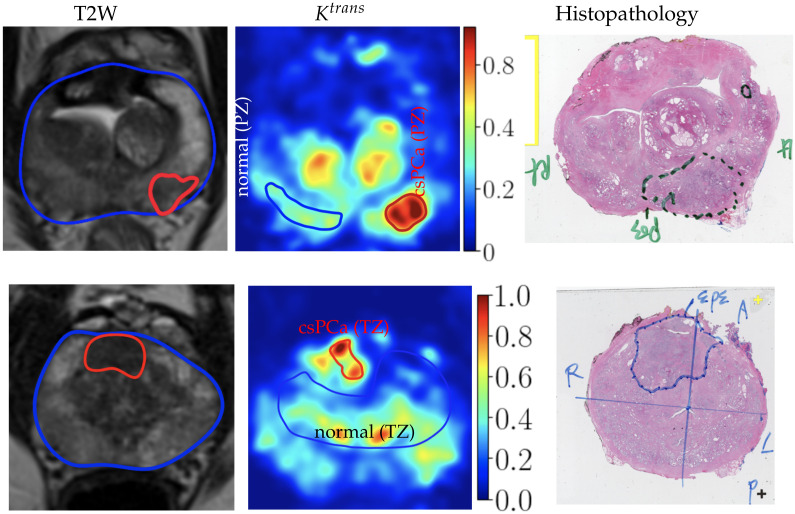
Illustration of how we annotated csPCa and normal tissue ROIs on $K^{trans}$ maps. Using the T2W annotations and the histopathological images as references, we annotated hyperintensity areas on $K^{trans}$ maps as lesions, and then we annotated normal tissue in the same zone on the $K^{trans}$ maps.

Due to severe misalignment in the prostate tumor delineation between T2W images and DCE [50], the annotations on T2W could not be propagated to $K^{trans}$. Instead, we annotated csPCa lesions on $K^{trans}$ maps with histopathological images as a reference. As shown in Figure 12, we first annotated hyperintensity regions as lesions (red ROIs) with T2W and histopathological images as references. To ensure a fair comparison that did not favor our method, the ROIs were annotated on $K^{trans}$ maps derived from NLLS with the standard Tofts model and Parker AIF and then propagated to other parameter maps (e.g., ktrans, $k_{ep}$, and λ maps derived from different methods). In addition to lesions, we also annotated the corresponding ROI of normal tissue (blue ROIs in Figure 12) in the same zone (transitional zone or peripheral zone) for contrast analysis. The ROI annotation could be biased due to being annotated on Ktrans maps. However, it is not biased toward any specific DCE-MRI analysis method, ensuring that the comparisons are fair.

After the annotation, ROI-averaged PK parameters are calculated for ROI-wise quantitative evaluations. For each patient, we annotate a representative lesion and a corresponding normal tissue. There are 135 lesions in the peripheral zone (PZ) and 47 lesions in the transitional zone (TZ).

**Figure 13:**
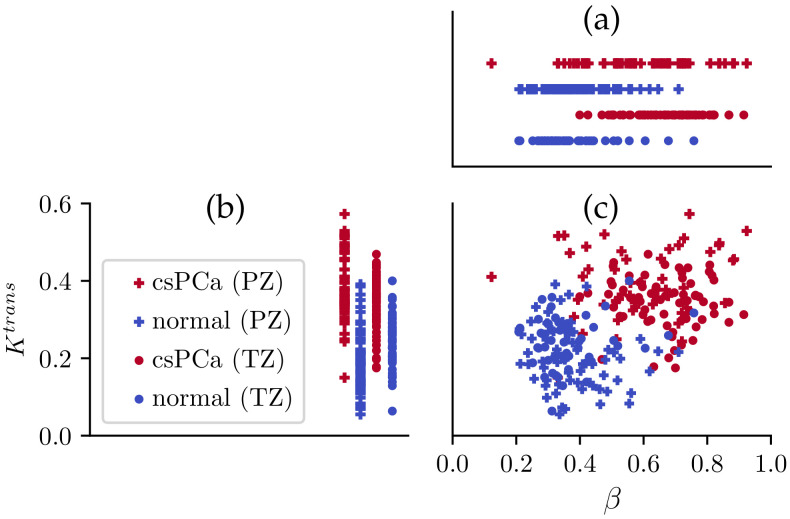
Scatter plot of ROI-averaged $K^{trans}$ and λ values in TZ and PZ. When applied individually, both λ (**a**) and $K^{trans}$ (**b**) can differentiate csPCa lesions from normal tissue, while $K^{trans}$ performs better. When applied jointly (**c**), the lesions and normal tissue can be better separated.

The scatter plot in Figure 13 depicts the $K^{trans}$ and λ values of csPCa and normal tissue in PZ and TZ. Not unexpectedly, as demonstrated in Figure 13a,b, csPCa ROIs generally have larger values for both $K^{trans}$ and λ. csPCa and normal tissue are more separable when simultaneously considering $K^{trans}$ and λ, as demonstrated in Figure 13c.

**Figure 14:**
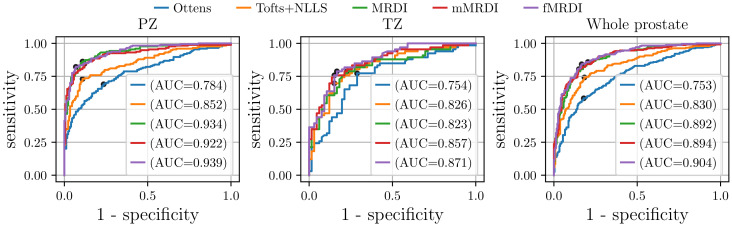
Specificity–sensitivity curves of csPCa detection in the peripheral zone (PZ), the transitional zone (TZ), and the whole prostate (PZ+TZ). Different colors represent different pharmacokinetic models. ‘Ours’ represents the fMRDI model with NN + refine curve fitting.

We applied the linear discriminant analysis (LDA) to quantitatively analyze the discrimination of csPCa from normal tissue in PZ and TZ. The ROI-averaged PK parameters ($K^{trans}$, $k_{ep}$, $t_{0}$, and λ) values derived from different methods were used for the classification of csPCa from normal tissue. The performance of each DCE-MRI analysis model was evaluated using 5-fold cross-validation, and the average results are reported. Sensitivity and specificity values were calculated from the cut-off points on the ROC curves by maximizing Youden’s index. The specificity-0sensitivity curves are shown in Figure 14, and the specificity, sensitivity, and AUC values are summarized in Table 3. In particular, in Figure 14, for ‘NLLS’, we employed the Tofts model with $K^{trans},k_{ep},t_{0}$ as the input to the LDA model. For ‘Ours’, we used our fMRDI model and $K^{trans},k_{ep},t_{0}$ as the input. And for ‘Ours (with λ)’, we used the fMRDI model with $K^{trans},k_{ep},t_{0}$, and λ as the input.

The results in Figure 14 and Table 3 demonstrate the following: (1) using the same PK parameters, our fMRDI model (‘Ours’) improves the csPCa detection performance compared to Ottens and NLLS. (2) the detection performance is further improved by using λ as additional input (‘Ours (with λ)’). The AUCs in Table 3 were compared via DeLong’s test with a 95% confidence interval, and the specificity and sensitivity were compared via the Chi-squared test (p<0.05).

**Figure 15:**
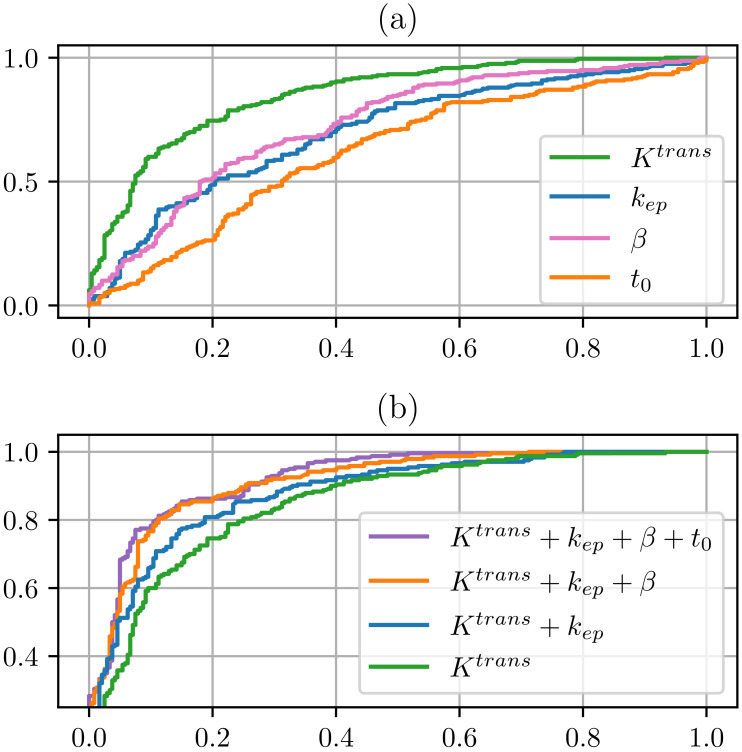
ROC curves from LDA analysis were generated for the entire prostate (PZ + TZ) using various combinations of PK parameters. (**a**) When analyzed individually, $K^{trans}$ exhibited the highest performance, followed by λ and then $k_{ep}$. Conversely, $t_{0}$ demonstrated the lowest performance and could only differentiate lesions from normal tissue to a limited extent. (**b**) When analyzed collectively, excluding $t_{0}$ barely impacted the performance, and the inclusion of λ moderately improved the performance.

Figure 15 demonstrates the ROCs of different combinations of PK parameters. When applied individually, $K^{trans}$ performed the best, followed by λ and $k_{ep}$. And $t_{0}$ can barely differentiate csPCa from normal tissue. When applied individually, the inclusion of λ noticeably improved the performance.

### 3.3. Validation with Digital Reference Objects

**Figure 16:**
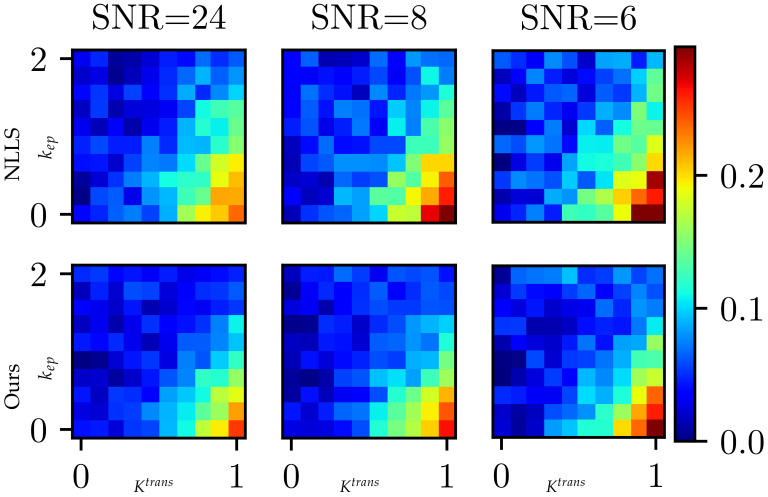
The fitting error of different methods under different noise levels using DROs. The heat maps show fitting errors at different $K^{trans}$ and $k_{ep}$ bins. The table summarizes the average errors at different noise levels.

Our two-phase inference pipeline is more resilient to noise because the neural network provides a stable initial state for subsequent iterative refinement. In this experiment, we tested the robustness of different methods against data noise using digital reference objects (DROs). The DROs are synthetical input data that are generated similarly to our training data using Equation (7) with two exceptions: (1) we used the plain Tofts model without λ for DRO synthesis for a fair comparison; (2) the parameters $K^{trans}$ and $k_{ep}$ were sampled from uniform distributions U(0,0.4) and U(0,2) to cover a wider range of possible inputs. The noise level of DROs was controlled via γ in Equation (7), whereas a larger γ indicates a higher level of noise. We synthesized 100K DROs that are grouped into 100=10×10 discrete bins according to their $K^{trans}$ and $k_{ep}$ values. Let $\hat{P}\in R^{2}=\left\{\hat{K^{trans}},\hat{k_{ep}}\right\}$ be the estimated parameter and *P* be the ‘ground-truth’, which was used to synthesize the corresponding DRO; the estimation error is calculated as follows: $error=\frac{{\left|\hat{P}-P\right|}^{2}}{{\left|P\right|}^{2}}$. We compared the fitting error of NLLS and our method under different noise levels in Figure 16. For example, the first bin comprises 1000 DROs with $K^{trans}$ ranging from 0 to 0.1 and $k_{ep}$ ranging from 0 to 0.2.

In the heat maps of Figure 16, we calculated the average estimation error within each bin. We conducted the paired sample t-test to verify that our method achieves lower fitting errors. And the *p*-values (p<0.001) confirmed the significance of our hypothesis. In general, the fitting errors surge with a higher level of noise. Under the same noise level, the fitting errors increase with the raising of $K^{trans}$ and $k_{ep}$. Our approach consistently provides more accurate results by reducing estimation errors, especially when dealing with higher levels of noise. This showcases the robustness and superiority of our method in handling noisy data.

## 4. Discussion

This study introduced the fast MRDI model and a deep learning-based, two-stage framework for efficient prostate DCE-MRI analysis. Existing quantitative DCE-MRI analysis methods rely on the time-consuming, iterative optimization of a pharmacokinetic (PK) model, e.g., the Tofts model [10]. PK modeling requires the characterization of arterial input functions (AIFs), and a population-based AIF is often assumed across patients. MR dispersion imaging (MRDI) [24] characterizes the intravascular dispersion of the contrast agent, yielding voxel-specific AIFs with various dispersion levels. The mMRDI [25] approximates the dispersion-applied AIFs with fewer dispersion-related parameters. The formulation of these models is highly nonlinear and poses difficulty in optimization.

In this study, we first introduced the fast MR dispersion imaging (fMRDI) model that simulates the dispersion-applied AIFs using a weighted sum of a slow and a fast AIF and that effectively forms location-specific AIFs with various dispersion levels, yielding estimates of the intravascular dispersion-related parameter λ. fMRDI is much simpler and easier to optimize compared to MRDI and mMRDI. Additionally, we proposed a deep-learning-based, two-stage framework for PK parameter estimation. We used a transformer-based neural network (NN) to perform a coarse estimation of the PK parameters, and then we used iterative NLLS to further refine the coarse estimation. The NN estimation essentially serves as an initialization for the subsequent NLLS, and our two-stage framework significantly speeds up and enhances the accuracy of PK modeling. A data-synthesis module was designed to generate synthetic data for neural network training. Two data-preprocessing modules were proposed to enhance the stability of the neural network.

Experimental results on our in-house clinical prostate MRI dataset demonstrated that the fMRDI model improves the fitting accuracy, and the dispersion parameter λ is able to improve the differentiation of csPCa from normal tissue. Our two-stage, deep learning-based method significantly accelerates the quantitative DCE-MRI analysis with more stable estimation.

There were several limitations in the current study. In the quantitative contrast analysis, the regions of interest (ROIs) were annotated on pharmacokinetic (PK) maps instead of being propagated from T2, potentially introducing bias in ROI annotation. Our data acquisition protocol did not include the first pass of the bolus, and the acquisition time was relatively short, which may not have been enough to measure the extra-vascular extracellular space, $v_{e}$. This poses challenges in extending our method to other organs, e.g., the liver. Future work will include employing advanced techniques such as neural differentiation equations for continuous-time DCE analysis, as well as extending and applying the proposed computational method to other DCE applications.

## 5. Conclusions

In conclusion, this study has made two contributions to improve prostate MR dispersion imaging: (i) Introducing the fMRDI model, which efficiently represents AIFs of different dispersion levels using a weighted-sum AIF. (ii) Developing a deep learning-based, two-stage method for estimating PK parameters. We believe the proposed techniques will improve the practical utility of DCE-MRI in oncological applications.

## Figures and Tables

**Table 1:** Squared errors are assessed by incrementally adding model components. The baseline model, labeled ‘NN + Parker’, represents the NN model without the proposed preprocessing or WS AIF.

NN + Parker AIF	0.6681±2.2015
+WS AIF	0.5982±1.9383
+Pyramid	0.5892±1.9012
+Sinusoidal	0.5801±1.8729
+Refine	0.4114±1.5181

**Table 2:** Squared errors, iterations required to converge, and per-patient times of different fitting methods and PK models. fMRDI achieves significantly lower errors compared to the Tofts model, and the two-stage fitting method also outperforms NLLS in both fitting errors and running time.

Fitting Method	Ottens	NLLS			NN + NLLS Refine		
PK Model	Tofts + Exp	Tofts + Parker	MRDI	fMRDI	Tofts + Parker	MRDI	fMRDI
Error	0.6723 ±2.2209	0.6184 ±1.9867	0.4272 ±1.6618	0.4261 ±1.5687	0.5917 ±2.1221	0.4175 ±1.5212	0.4114 ±1.5181
Iterations	N/A	200	300	200	20	50	30
Time (per-patient)	109 s	480 s	644 s	480 s	71 s	115 s	176 s

**Table 3:** Sensitivity, specificity, and AUC values of different methods in the PZ, the TZ, and the whole prostate. The sensitivity and specificity values are calculated by maximizing Youden’s index.

Method	Ottens [38]			NLLS+Tofts+Parker			MRDI			fMRDI (Ours)		
Zone	PZ	TZ	PZ+TZ	PZ	TZ	PZ+TZ	PZ	TZ	PZ+TZ	PZ	TZ	PZ+TZ
AUC	0.784	0.754	0.753	0.852	0.826	0.830	0.934	0.823	0.892	0.939	0.871	0.904
1 - specificity	0.236	0.288	0.179	0.109	0.152	0.183	0.109	0.227	0.204	0.069	0.167	0.167
Sensitivity	0.690	0.773	0.583	0.730	0.697	0.742	0.862	0.788	0.858	0.822	0.788	0.842

## Data Availability

The dataset collected from our institution is currently not publicly available since the IRB only approves its employment for internal usage. The data might be available for research purposes upon reasonable request or for institutional collaborations. Please contact Kyunghyun Sung (ksung@mednet.ucla.edu) for any dataset-specific requests.
